# Implementation of an electronic interface for the documentation of pediatric rheumatology medical records improves physician and patient utilization of time in clinic

**DOI:** 10.1186/1546-0096-10-S1-A15

**Published:** 2012-07-13

**Authors:** Jennifer MP Woo, Miriam F Parsa, Gil Amarilyo, Nasim Afsar-manesh, Kerry Gallagher, Ornella J Rullo, Deborah K McCurdy

**Affiliations:** 1 UCLA, Manhattan Beach, CA, USA; 2 UCLA, Los Angeles, CA, USA

## Purpose

Paper clinical notes (PCN) have been an enduring tool in clinical medicine, but are slowly being replaced by electronic medical records (EMR) in a nationwide effort to improve clinic efficiency, to enhance interdepartmental communication, and to minimize medical errors. Our healthcare center utilizes a transitional EMR that requires outpatient clinic physicians to document patient visits on PCN and to submit them to an external department for upload to the center-wide EMR. This process generates a lag period, ranging from 1 day to 3 weeks, during which PCN are inaccessible electronically. If notes are unavailable via EMR at subsequent visits, physicians are forced to search through paper charts, which may be located in clinic or at a satellite office, thus impeding clinic workflow and lengthening patient wait time. In response, we designed, created, and implemented an EMR interface or electronic note (EMRI) that mimics the prior paper template. Physicians use a portable laptop computer in the exam room and directly upload their findings to the patient’s center-wide EMR, reducing the time required for follow-up charting. We are evaluating the workflow of the pediatric rheumatology outpatient clinics and assessing patient perceptions as we transition from PCN to a complete EMR system.

## Methods

Our EMRI is a Microsoft Excel form that utilizes VBA macros to aid the physician in documenting an accurate account of the patient’s present health. Clinic efficiency was evaluated by monitoring the length of time necessary to locate previous notes, time physicians spent with each patient, patient wait time, time dedicated to follow-up charting, and time required for clinic notes to be uploaded to the EMR. Patients or their parents were asked, via paper survey, to anonymously assess the quality of care received and their perception of EMRI use in clinic; observations will continue until our EMRI is fully integrated into clinical practice.

## Results

Visits documented using the EMRI presented a significantly greater patient-physician interaction time (69% vs. 53%; p = 0.04) and significantly less patient wait time compared to those documented using PCN (31% vs. 47%; p = 0.04) with no change in overall appointment length. On average, physicians spent 70 minutes less on follow up charting at the end of clinic when using the EMRI (69 minutes) compared to PCN (140 minutes). EMRI utilization reduced the average time required for clinical documents to be available on center-wide EMR by 89% (0.8 days vs. 7.4 days; p < 0.0000001). In addition, 100% of EMRI notes were available center-wide within 4 days of the patient’s visit compared to 21 days required for 100% of PCN (p < 0.0001). Currently, 94% of patients/parents surveyed (n = 40) were receptive to EMRI use and 69% believed that an EMRI would benefit the patient’s quality of care (30% were neutral regarding the benefits of an EMRI).

## Conclusion

When transitioning from PCN to EMRI documented notes in an academic outpatient pediatric rheumatology practice, clinic efficiency, direct patient-physician interaction, and patient perception of quality of care are maintained and potentially optimized while continuing to provide superior medical care.

## Disclosure

Jennifer M.P. Woo: None; Miriam F. Parsa: None; Gil Amarilyo: None; Nasim Afsar-manesh: None; Kerry Gallagher: None; Ornella J. Rullo: None; Deborah K. McCurdy: None.

